# Analysis of Uncertainty in a Middle-Cost Device for 3D Measurements in BIM Perspective

**DOI:** 10.3390/s16101557

**Published:** 2016-09-22

**Authors:** Alonso Sánchez, José-Manuel Naranjo, Antonio Jiménez, Alfonso González

**Affiliations:** 1 University Centre of Mérida, University of Extremadura, 06800 Mérida, Spain; agg@unex.es; 2 Polytechnic School, University of Extremadura, 10003 Cáceres, Spain; jnaranjo@unex.es; 3 Development Area, Provincial Council of Badajoz, 06071 Badajoz, Spain; ajimenez.atm@dip-badajoz.es

**Keywords:** photogrammetry, point clouds, uncertainty, constrained least squares adjustment, middle-cost device

## Abstract

Medium-cost devices equipped with sensors are being developed to get 3D measurements. Some allow for generating geometric models and point clouds. Nevertheless, the accuracy of these measurements should be evaluated, taking into account the requirements of the Building Information Model (BIM). This paper analyzes the uncertainty in outdoor/indoor three-dimensional coordinate measures and point clouds (using Spherical Accuracy Standard (SAS) methods) for Eyes Map, a medium-cost tablet manufactured by e-Capture Research & Development Company, Mérida, Spain. To achieve it, in outdoor tests, by means of this device, the coordinates of targets were measured from 1 to 6 m and cloud points were obtained. Subsequently, these were compared to the coordinates of the same targets measured by a Total Station. The Euclidean average distance error was 0.005–0.027 m for measurements by Photogrammetry and 0.013–0.021 m for the point clouds. All of them satisfy the tolerance for point cloud acquisition (0.051 m) according to the BIM Guide for 3D Imaging (General Services Administration); similar results are obtained in the indoor tests, with values of 0.022 m. In this paper, we establish the optimal distances for the observations in both, Photogrammetry and 3D Photomodeling modes (outdoor) and point out some working conditions to avoid in indoor environments. Finally, the authors discuss some recommendations for improving the performance and working methods of the device.

## 1. Introduction

The three-dimensional modeling of an object begins with the required data acquisition process for the reconstruction of its geometry and ends with the formation of a virtual 3D model that can be viewed interactively on a computer [1]. The information provided by the display of these models makes its application possible for different uses [2], such as the inspection of elements, navigation, the identification of objects and animation, making them particularly useful in applications such as artificial intelligence [3], criminology [4], forestry applications [5,6], the study of natural disasters [7,8], the analysis of structural deformation [9,10], geomorphology [11,12] or cultural heritage conservation [13,14].

In particular, the generation of point clouds and 3D models has important applications, especially in Building Information Modeling (BIM). This digital representation of the physical and functional characteristics of the buildings serves as an information repository for the processes of design and construction, encouraging the use of 3D visualizations [15]. In the future, devices could include different types of sensors to capture all kind of information for BIM applications. In addition, important technological advances in automated data acquisition has led to the production of more specific models tailored to Historic Building Information Modeling (HBIM) for the preservation of historical or artistic heritage [16,17].

In recent years, different techniques have been developed to acquire data [18]. On the one hand, there are active measurement techniques, carrying out modeling based on scans (range-based modeling), which uses instruments equipped with sensors that emit a light with a structure defined and known by another sensor that has to capture it [19]. On the other hand, there are passive measurement techniques, with modeling based on images (image-based modeling), which use optical or optical-electronic capture systems for extracting geometric information in the construction of 3D models [19]. The former uses different types of laser scanners, while the latter employs photogrammetric or simple conventional cameras. In each case, specific software for data processing is used.

One of the most important geometric aspects is the verification of the accuracy and reliability of measurements with which data are acquired and the resulting 3D models are obtained, since, according to the tolerances and maximum permissible errors required for the use of certain models, for example in BIM working environment, the final accuracy and reliability obtained with a specific device will determine its suitability for certain works [20]. Many studies have carried out such analysis for active measurement techniques [21,22,23] as in the case of passive measurement techniques [4,24,25]. These are deduced in the first case for objects of medium format, with the use of handheld laser scanners, where an accuracy up to 0.1 mm can be achieved [26]; in the second case, using techniques of automated digital photogrammetry, precision is of the order of about 5 mm [27], but with the advantage of a smaller economic cost.

There are instruments equipped with a low-cost sensor on the market: the David laser scanner [28], Microsoft Kinetic v1 and v2 sensors, and RGB-D cameras. These cameras are easy to manage and they are being used for applications that require a precision of about 5 mm to a measured distance of 2 m [29]. There are also middle-cost devices, based on structured light technology, such as the DPI-8 Handheld Scanner (from DotProduct LLC, Boston, MA, USA) and the FARO Freestyle^3D^ Scanner (FARO, Lake Mary, FL, USA).

Nowadays, there are new projects that are trying to enter the market using instruments based on a smartphone or a tablet including a range imaging camera and special vision sensor, which are user-friendly, affordable and offer accuracy for a wide range of applications. These include Google’s Tango project from 2014, the Structure Sensor from Occipital from 2015 and EyesMap (EM) carried by e-Capture Research and Development from 2014.

Nonetheless, one of the main problems encountered when performing 3D modeling is to determine the accuracies obtained with these devices, especially when taking into account the rate of information uptake and the intended product. Normally, the two products we are trying to obtain are geometric models and 3D point clouds. The first is used to describe the shape of an object, by means of an analytical, mathematical and abstract model. The second produces very dense and elaborate coordinate data points for the surfaces of a physical object [30,31]. For this reason, one objective of this paper is to perform an analysis of the accuracy of the EM, in two modes of data capture: (1) Photogrammetry to get 3D point coordinates; and (2) Photomodeling to get 3D point cloud and the color of the object observed.

This accuracy was evaluated by comparison with the EM measurements and the data acquired by a Total Station. On the other hand, operator error was estimated by comparison with the coordinates of symmetrical target centers measured by EM and by a Scanstation. Additionally, to investigate the feasibility of coordinates, measurements and point cloud acquisition from a BIM perspective, further evaluation was performed in reference to the guidelines of the GSA for BIM Guide for 3D Imaging [32].

## 2. Materials and Methods

**Figure 1:**
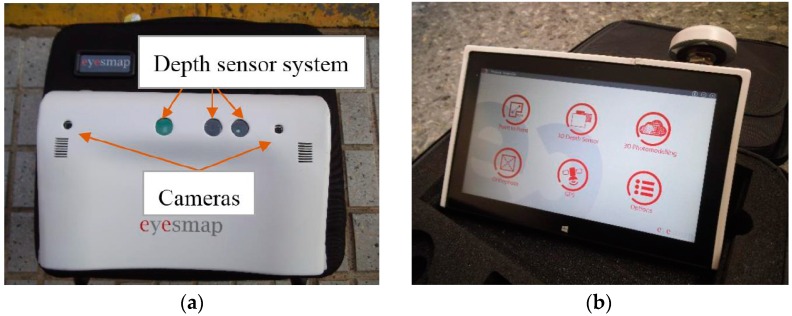
EyesMap (EM): (**a**) back; and (**b**) front.

This study was conducted with an EM tablet from e-capture Research & Development Company. It has dimensions of 303 × 194 × 56 mm^3^, a weight of 1.9 kg and a screen of 11.6 inches. The device has a processor Intel Core i7, 16 gigabytes of RAM and runs on the Windows 8 operating system. It has an Inertial Measurement Unit and a GNSS system, which allow for measuring speed, orientation and gravitational forces, as well as the positioning of the instrument in real time. To capture the three-dimensional information, on the back of the tablet (Figure 1), there is a depth sensor and two cameras with a focal length of 2.8 mm and a 13 megapixel resolution, that form a base line of 230 mm, with a field of view up to 67°.

The beta version of EM costs around €9500. The basic principle of operation is based on photogrammetry techniques, which reconstruct a scene in real time. The precision indicated by the manufacturer (Table 1) for both measurement modes are:

Precisely in order to achieve the precisions expressed in the previous table, the recommendations of the manufacturer for the Photogrammetry measurement are: (1) take at least 2 pictures; (2) 80% overlap/2 pictures; and (3) capture in parallel or convergent. In the case of measurement by 3D Photomodeling, the same recommendations apply, but take at least five pictures instead of two. EM uses a computer vision approach based on general method of Photogrammetry [33].

In this sense, obtaining coordinates ($X_{P},Y_{P},Z_{P})$, is computed by Digital Image Correlation (DIC). In this way, 3D cloud points are achieved to a very high density from the surface of the studied object, moreover, storing color information (RGB). The calculation process of the coordinates of the points that compose the cloud, from a pair of oriented pictures is carried out by the method of triangulation [34].

The continuous evolution of algorithms that perform DIC has been reaching very high levels of precision and automation. Currently, the most effective are Structure from Motion (SFM) and the algorithms of Reconstruction in 3D in high density named Digital Multi-View 3D Reconstruction (DMVR) which produce 3D models of high precision and photorealistic quality from a collection of disorganized pictures of a scene or object, taken from different points of view [35].

### 2.1. EM Workflow

The processes of calibration and orientation of cameras are implemented in the EM software. The orientation of pictures can be done in three ways: (1) automatic orientation, matching homologous points that the system finds in both pictures; (2) manual orientation, in which the user chooses at least 9 points in common in both pictures; and (3) automatic orientation by means of targets, which require the existence of at least 9 asymmetrical targets in common. The latter one offers major precision and requires a major processing time. The information obtained can also be viewed in real dimension by means of the target named the Stereo target. EM offers the following options: Photogrammetry, 3D Photomodeling, 3D Modeling with Depth Sensor and Orthophoto. Photogrammetry allows for measuring coordinates, distances and areas between points, as well as exporting its coordinates in different formats (*.txt and *.dxf) so other computer aided design programs can be used. 3D Photomodeling and 3D Modeling with Depth Sensor allow 3D point clouds with XYZ and color information (PLY and RGB formats respectively), from an object. However, modeling with the support of the depth sensor is restricted for indoor work, offering less precise results than the 3D Photomodeling. The last gives an orthophotograph of the work area.

**Figure 2:**
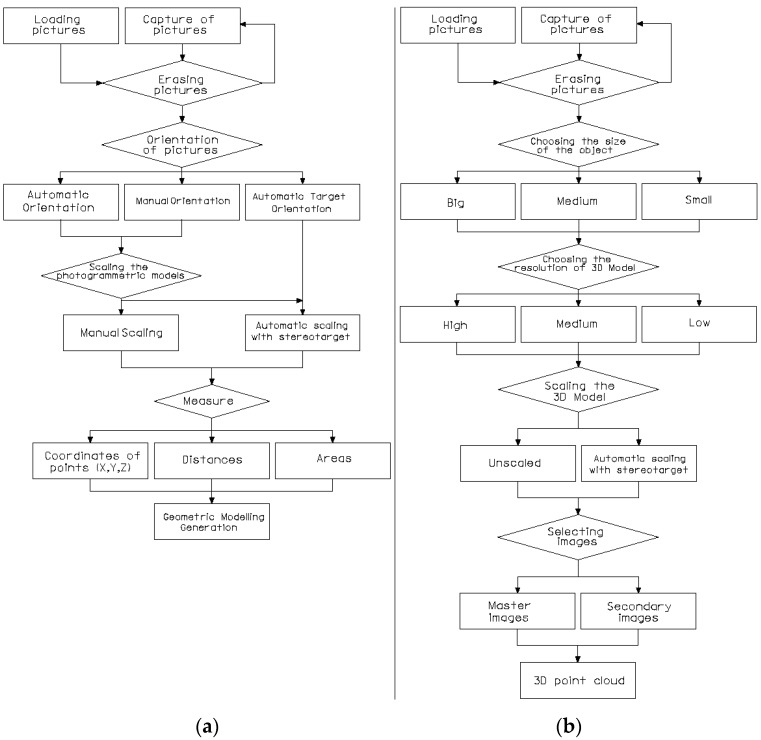
Workflow of EM measurement: (**a**) Photogrammetry; and (**b**) 3D Photomodeling.

In Photogrammetry (Figure 2a), pictures can be either captured or loaded. Secondly, pictures have to be managed and the desired pictures selected. Thirdly, we can choose: (1) automatic orientation; (2) manual orientation; or (3) automatic target orientation, in order to achieve the relative orientation of the pictures. In this regard, an automatic scale is made by means of automatic target orientation and the Stereo target is used. After this, the following measurements can be obtained: (1) coordinates of points; (2) distances; or (3) areas. Finally, the geometric model is obtained.

In 3D Photomodeling (Figure 2b), pictures are managed in the same way as Photogrammetry. Secondly, the object to be measured according to its size is selected: small if dimensions are less than one meter, medium-sized if the dimensions are below 10 m and large for all other dimensions. Consequently, high, medium or low resolution must be selected. The final model will be scaled or unscaled by means of the Stereo target. After this, the master picture can be selected.

In each of these four options, different working procedures are followed, depending on capture methodology, shooting planning, and the size and characteristics of the object to measure. Figure 2 shows the two options that were used in this study.

### 2.2. Data Acquisition Systems

**Figure 3:**
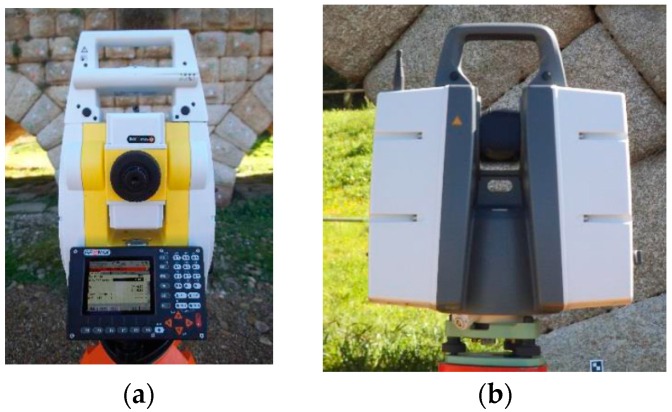
Used equipment: (**a**) Geomax Zoom 80 high precision Total Station; and (**b**) Leica ScanStation P30.

This work is going to determine the instrumental errors of EM for two of the measurement options available: (1) Photogrammetry; and (2) 3D Photomodeling. To achieve it, we have resorted to two other, more precise, measurement instruments [28,31,35]. The Geomax Zoom 80 (GeoMax AG, Widnau, Switzerland) high precision Total Station, with a standard deviation of 2″ (0.6 mgon) for the angular measures and 2 mm ± 2 ppm for the distance measurements (Figure 3a), and the Scanstation Leica P30 (Leica Geosystems AG, Heerbrug, Switzerland), with a standard deviation in the 3D position of 3 mm (Figure 3b).

**Figure 4:**
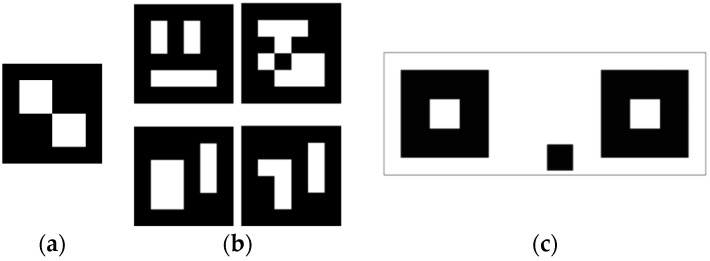
Targets provided by EM: (**a**) symmetric target; (**b**) asymmetric targets; and (**c**) stereo target.

Regarding Photogrammetry, the coordinates of the center of the symmetrical targets (Figure 4) were measured by EM, on a canvas 1, 2, 3, 4, 5 and 6 m away. Subsequently, these measurements and the measurements obtained by means of the high precision Total Station were compared.

Symmetrical targets were used with asymmetric targets and the Stereo target. The asymmetric targets served for the automatic orientation of the stereoscopic pairs, because this is the most accurate way according to the manufacturer. The Stereo target was also used to scale the obtained measurements.

Regarding the measurement by 3D Photomodeling, high-resolution point clouds were achieved by EM from 1–6 m to the canvas. Subsequently, the centers of symmetrical targets were measured from the point clouds by means of CloudCompareV2 and they were compared with the coordinates obtained by the high precision Total Station. In any case, no point of the clouds obtained by EM coincides exactly with the center of a target and it is necessary to locate and measure the closest point to this center (not the real center) using CloudCompareV2. On the other hand, only the coordinates of the targets that could be correctly identified were measured.

### 2.3. Data Processing

The coordinates measured by EM (*x*, *y*, *z*); and those obtained by the Total Station and the Scan station (*X*, *Y*, *Z*) are geo referenced on different coordinate systems. To be able to compare them, the coordinates obtained by EM were transformed to the coordinate system provided by the Total Station. The transformation that was used was the so-called Helmert or 7 parameters. The three steps of this transformation are: (1) three rotation angles (Ω, Φ, Κ); (2) three translations (*Tx*, *Ty*, *Tz*); and (3) a change of scale (λ), which except for the last step were calculated using the EM coordinates system. Both systems of coordinates were parallel. Through the translations, both systems would have the same origin of coordinates. Finally, the scale factors of both systems of coordinates have the same measurement units. Nonetheless, the application of the scale factor may alter the measurements [36], which was not applied for this reason.

(1) $$\left[\begin{array}{c} X\\ Y\\ Z \end{array}\right]=\left[\begin{array}{ccc} a_{11} & a_{12} & a_{13}\\ a_{21} & a_{22} & a_{23}\\ a_{31} & a_{32} & a_{33} \end{array}\right]\left[\begin{array}{c} x\\ y\\ z \end{array}\right]+\left[\begin{array}{c} T_{X}\\ T_{Y}\\ T_{Z} \end{array}\right]$$

where:

(2) $$\begin{array}{ccc} a_{11}=\cos\Phi \cos Κ & a_{12}=-\cos\Phi \sin Κ & a_{13}=\sin\Phi \\ a_{21}=\cos\Omega \sin Κ+\sin\Omega \sin\Phi \cos Κ & a_{22}=\cos\Omega \cos Κ-\sin\Omega \sin\Phi \sin Κ & a_{23}=-\sin\Omega \cos\Phi \\ a_{31}=\sin\Omega \sin Κ & a_{32}=\sin\Omega \cos Κ+\cos\Omega \sin\Phi \sin Κ & a_{33}=\cos\Omega \cos\Phi \end{array}$$

The equations were linearized for a point P by means of the development in Taylor series to the first term:

(3) $$X_{P}={\left(X_{P}\right)}_{0}+{\left(\frac{\partial X_{P}}{\partial \Omega }\right)}_{0}d\Omega +{\left(\frac{\partial X_{P}}{\partial \Phi }\right)}_{0}d\Phi +{\left(\frac{\partial X_{P}}{\partial Κ}\right)}_{0}dΚ+{\left(\frac{\partial X_{P}}{\partial T_{X}}\right)}_{0}+dT_{X}+{\left(\frac{\partial X_{P}}{\partial T_{Y}}\right)}_{0}dT_{Y}+{\left(\frac{\partial X_{P}}{\partial T_{Z}}\right)}_{0}dT_{Z}$$

(4) $$Y_{P}={\left(Y_{P}\right)}_{0}+{\left(\frac{\partial Y_{P}}{\partial \Omega }\right)}_{0}d\Omega +{\left(\frac{\partial Y_{P}}{\partial \Phi }\right)}_{0}d\Phi +{\left(\frac{\partial Y_{P}}{\partial Κ}\right)}_{0}dΚ+{\left(\frac{\partial Y_{P}}{\partial T_{X}}\right)}_{0}+dT_{X}+{\left(\frac{\partial Y_{P}}{\partial T_{Y}}\right)}_{0}dT_{Y}+{\left(\frac{\partial Y_{P}}{\partial T_{Z}}\right)}_{0}dT_{Z}$$

(5) $$Z_{P}={\left(Z_{P}\right)}_{0}+{\left(\frac{\partial Z_{P}}{\partial \Omega }\right)}_{0}d\Omega +{\left(\frac{\partial Z_{P}}{\partial \Phi }\right)}_{0}d\Phi +{\left(\frac{\partial Z_{P}}{\partial Κ}\right)}_{0}dΚ+{\left(\frac{\partial Z_{P}}{\partial T_{X}}\right)}_{0}+dT_{X}+{\left(\frac{\partial Z_{P}}{\partial T_{Y}}\right)}_{0}dT_{Y}+{\left(\frac{\partial Z_{P}}{\partial T_{Z}}\right)}_{0}dT_{Z}$$

On the basis of the linearized equations of general expression and knowing the coordinates in both systems of at least two points, the following equations were formed:

(6) $$r_{11}^{n}d\Omega +r_{12}^{n}d\Phi +r_{13}^{n}dΚ+r_{14}^{n}dT_{X}+r_{15}^{n}dT_{Y}+r_{16}^{n}dT_{Z}=X_{n}-{\left(X_{n}\right)}_{0}$$

(7) $$r_{21}^{n}d\Omega +r_{22}^{n}d\Phi +r_{23}^{n}dΚ+r_{24}^{n}dT_{X}+r_{25}^{n}dT_{Y}+r_{26}^{n}dT_{Z}=Y_{n}-{\left(Y_{n}\right)}_{0}$$

(8) $$r_{31}^{n}d\Omega +r_{32}^{n}d\Phi +r_{33}^{n}dΚ+r_{34}^{n}dT_{X}+r_{35}^{n}dT_{Y}+r_{36}^{n}dT_{Z}=Z_{n}-{\left(Z_{n}\right)}_{0}$$

Expressing the system of equations in matrix form:

(9) $$\left[\begin{array}{cccccc} r_{11}^{n} & r_{12}^{n} & r_{13}^{n} & r_{14}^{n} & r_{15}^{n} & r_{16}^{n}\\ r_{21}^{n} & r_{22}^{n} & r_{23}^{n} & r_{24}^{n} & r_{25}^{n} & r_{26}^{n}\\ r_{31}^{n} & r_{32}^{n} & r_{33}^{n} & r_{34}^{n} & r_{35}^{n} & r_{36}^{n} \end{array}\right]\left[\begin{array}{c} d\Omega \\ d\Phi \\ dΚ\\ dT_{X}\\ dT_{Y}\\ dT_{Z} \end{array}\right]-\left[\begin{array}{c} X_{n}-{\left(X_{n}\right)}_{0}\\ Y_{n}-{\left(Y_{n}\right)}_{0}\\ Z_{n}-{\left(Z_{n}\right)}_{0} \end{array}\right]=\left[\begin{array}{c} V_{X_{n}}\\ V_{Y_{n}}\\ V_{Z_{n}} \end{array}\right]$$

Applying the adjustment by least squares, the system of equations is solved and 6 transformation parameters were obtained (Ω, Φ, Κ, *Tx*, *Ty*, and *Tz*). Nevertheless, half of the coordinates of the center of the symmetrical measured targets were used. These were called Transformation Points. Subsequently, with the transformation parameters obtained, the other half of the coordinates of the center of symmetrical targets measured were transformed from the system of coordinates of EM to the system of coordinates of Total Station. The resulting Validation Points have two sets of coordinates in the coordinate system established by the Total Station: (1) coordinates transformed to the Total Station coordinate system; from the measured performed by EM; and (2) coordinates of reference directly measured by Total Station.

### 2.4. Uncertainty Assessment

**Figure 5:**
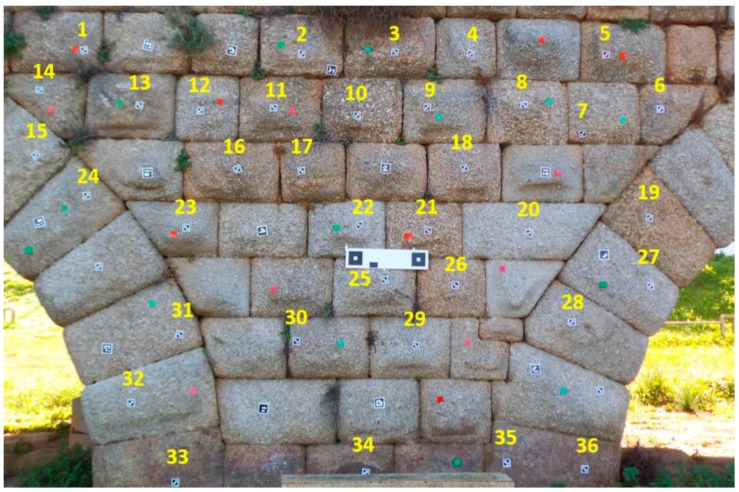
Used targets.

**Figure 6:**
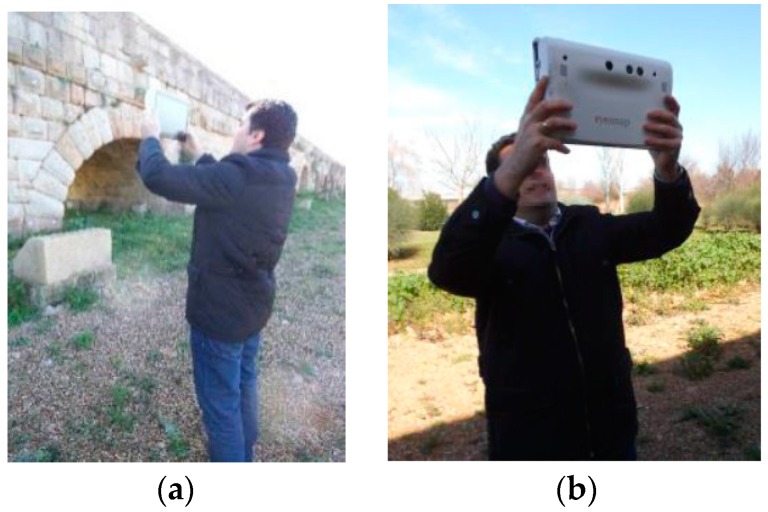
Data capture using EM: (**a**) front view; and (**b**) back view.

The measurements were made at the Roman Bridge in Merida (Spain), on a canvas of approximately 6 × 5 m^2^ (Figure 5 and Figure 6). This bridge, being of granite, presents an optimal texture for automatic correlation of images. EM was evaluated according to how correctly it measured elements placed at different depth levels.

**Figure 7:**
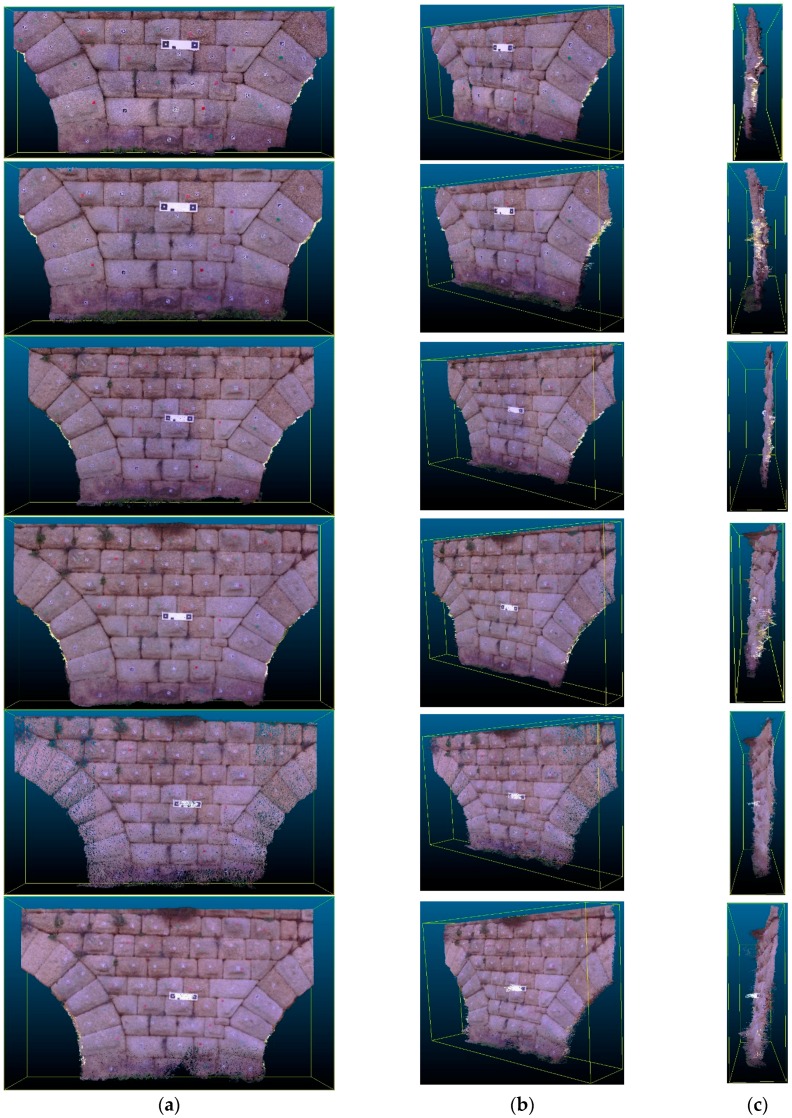
3D point clouds obtained by EM for 1, 2, 3, 4, 5 and 6 m, from up to down: (**a**) front view; (**b**) middle-side view; and (**c**) right side view.

The metric quality of measurements obtained by EM was evaluated using the method proposed by Hong et al. [31]. The three-dimensional coordinate measurements and point clouds obtained by EM (Figure 7) were compared to a set of Total Station point measurements used as reference points. In the mapping accuracy assessment, comparisons were based on identifiable target centers. These targets were distributed across the canvas. The accuracy assessment was based on the well-distributed and clearly identifiable point targets. A number of reference points were measured for each test side. In addition, using as a reference the tolerances established in the guidelines of the GSA for BIM Guide for 3D Imaging [32], the viability and acceptability of this measurement device for BIM generation was determined. According to [32], tolerance is the dimensional deviation allowed as error from the true value in the specified coordinate frame. The true value is a measurement obtained by other means.

Firstly, the precision of the measurements made by EM by Photogrammetry and 3D Photomodeling are evaluated through the Euclidean average distance error ($\delta_{avg}$).

(10) $$\delta_{avg}=\frac{1}{n}\sum_{i=1}^{n}\left|Ra_{i}-T-b_{i}\right|$$

where $a_{i}$ corresponding to the measurement is carried out by EM for the *i*-th check point in one case by Photogrammetry and in the other case by 3D Photomodeling and $b_{i}$ is the measurement made for the same point by Total Station. In addition, the rotation and translation parameters of the 3D Helmert transformation are *R* and *T*, respectively. Note that scale was not considered in this transformation [37].

Secondly, the corresponding average error is calculated, together with the error vectors in the *x*, *y* and *z* directions. The Root Mean Square Error (RMSE) is then calculated to assess the quality of the points captured by EM and measured by means of photogrammetry and 3D Photomodeling.

(11) $$\mathrm{RMSE}=\sqrt{\frac{1}{n}{\sum_{i=1}^{n}\left(a_{i}^{t}-b_{i}\right)}^{2}}$$

where $a_{i}^{t}$ shows the point transformed to the coordinates of the Total Station.

Thirdly, the quality of the points measured by EM is also assessed by calculating the Spherical Accuracy Standard (SAS). The SAS, which represents the spherical radius of a 90% probability sphere [38], is defined as

(12) $$\mathrm{SAS}=2.5\times 0.3333\times \left({\mathrm{RMSE}}_{x}+{\mathrm{RMSE}}_{y}+{\mathrm{RMSE}}_{Z}\right)$$

This represents a positional accuracy of the coordinates obtained by Photogrammetry and the point cloud obtained by 3D Photomodeling with a 90% confidence level. The calculation of errors was repeated for the measurements carried out by EM from 1–6 m to the object measured by Photogrammetry.

## 3. Results

Results were tabulated (Table 2 and Table 3) and shown in graphs. Different points were chosen in each case (3D Photomodeling and Photogrammetry modes) depending on the correct identification of target centers by the operator.

**Figure 8:**
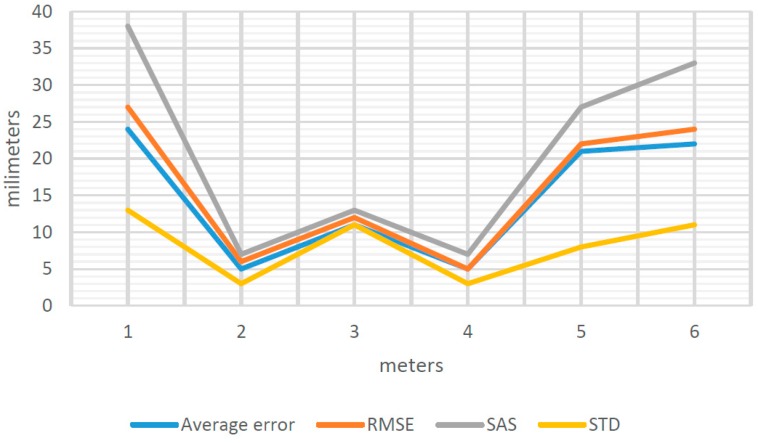
Average error, RMSE, SAS and STD for 1–6 m.

Similarly, there are estimates for observation distances from 1 to 6 m, obtaining the general results shown in Figure 8.

The value of Average Error, RMSE, SAS and STD (Figure 8) varied depending on the distance of separation between the object to be measured and the position from which we perform data capture by means of EM.

Nonetheless, as shown in Figure 8, error does not increase progressively as the separation distance increases. In fact, the optimum separation distances are 2, 3 and 4 m and not 1 m, as could be supposed. At 1, 5 and 6 m, the errors increase considerably.

The results obtained demonstrate that geometric models from between 2 and 4 m of distance to the object measured, satisfy the requirements of the GSA for BIM Guide for 3D Imaging [32] (Section 2.3. types of deliverables from 3D data) for projects in urban design, architectural design, room space measurement, historic documentation, renovation (level 2) and above ceiling condition capture. Subsequently, the quality of the point clouds obtained by EM by Photomodeling was evaluated. The point clouds were obtained from 1–6 m to the measured object. However, it was not possible to obtain errors for 5 and 6 m, since the low density of the mesh does not allow for correctly identifying the centers of the symmetrical targets. As a result, it was impossible to measure the coordinates of these targets.

As before, the measurements carried out by EM and measurements made by 3D Photomodeling are evaluated through the Euclidean average distance error ($\delta_{avg}$) (see Equation (10)).

**Figure 9:**
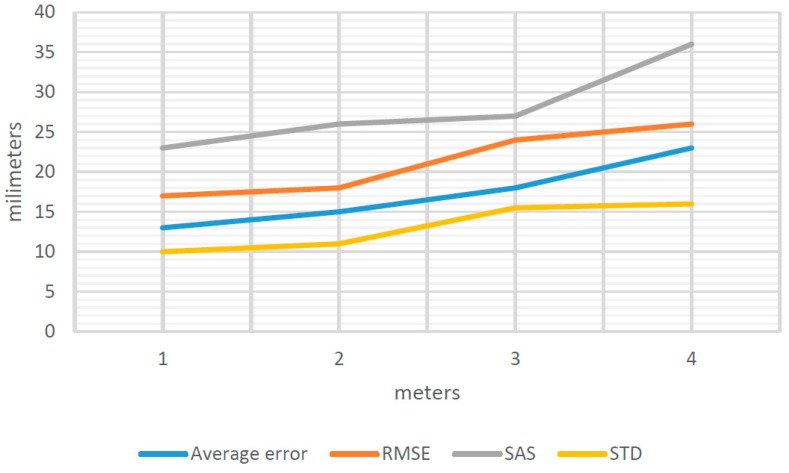
Average error, RMSE, SAS and STD for 1, 2, 3 and 4 m.

Similarly, there are estimates for distances of observation from 1 to 4 m, producing the general results that are shown in Figure 9.

The value of Average Error, RMSE, SAS and STD (Figure 9) varies depending on the distance of separation between the object to be measured and the position from which we perform data capture by EM.

As shown in Figure 9, error increases in proportion to the increase in separation distance from the object being measured. Therefore, the most appropriate distance for taking measurements is 1 m.

These errors show that point clouds between 1 and 4 m of distance from the measured object satisfy the requirements of the GSA for BIM Guide for 3D Imaging [32] for level 1 projects, urban design and historic documentation.

Nonetheless, errors for measurements obtained by both Photogrammetry and 3D Photomodeling are influenced by the operator error. This error is produced by the visual acuity of the operator, when the operator identifies a dot that appears in each picture. The identification of these types of point is done for different purposes, such as the adjustment of photographic pairs and the generation of geometric models. The estimate of this error allows evaluation of their influence on the previously obtained errors. To estimate error, the centers of symmetrical targets were identified at the 3D point clouds achieved by the Scanstation (only considering targets with a point measured close to their centers), the coordinates are measured and these are compared with the coordinates measured by Total Station, since these data are correct and associated errors are known.

The differences between coordinates are used to calculate the error for the vectors *x*, *y*, *z*. Error for each target is measured. In this case, we use the average distance of separation from the measured object, 3 m, in order to determine the standard deviation for the point cloud (Table 4). In this manner, the standard deviation of the measurements for the targets $STD_{T}$ is equal to 11 mm.

In addition, $STD_{T}$ is related to: (1) the standard deviation for the Scanner Station *STD_SC_* = ±3 mm in *X*, *Y*, *Z* coordinates; (2) the standard deviation for the Total Station *STD_ST_* = 2 mm ± 2 ppm and 2″ (0.6 mgon) also supplied by the manufacturer; and (3) the standard deviation of the operator $STD_{OP}$ when the operator measure the targets:

(13) $$STD_{T}=\sqrt{STD_{SC}{}^{2}+STD_{ST}{}^{2}+STD_{OP}{}^{2}}$$

The estimation of the error committed by the operator in the identification of the targets, in this case, is equal to 10 mm (Table 5). Likewise, if we take into account the standard deviation for measurements by Photogrammetry $STD_{PH}$ and Photomodelling $STD_{CP}$ (Figure 8 and Figure 9) and $STD_{OP}$ estimated previously, it was observed (Table 5) that there is a huge influence for this error on the measures carried out.

In this respect, the estimated error of the operator is roughly 91% of the total error measured by Photogrammetry and 62% when we measure with 3D Photomodeling. Note that the color of targets should be in black and white, since when we carried out tests with red and green targets, the error estimate for the operator was even higher.

## 4. 3D Modeling of Indoor Environments with EM

The instrument under study (EM) allows obtaining 3D models inside buildings and structures (indoor). For this, the manufacturer recommends using the option of working with the depth sensor system of the instrument (Figure 1), with which we can create a complete and scaled point cloud of an indoor environment in real time, with an object to device distance less than 4 m.

**Figure 10:**
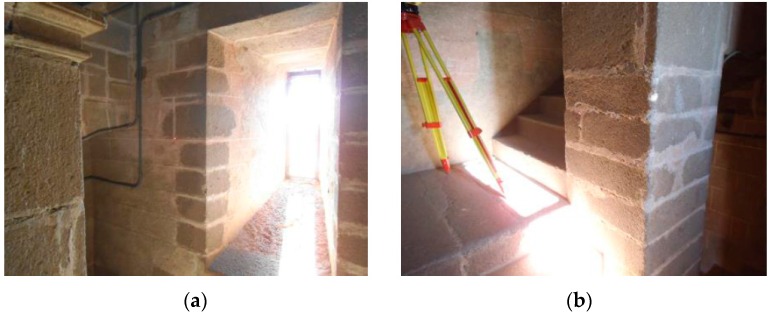
Walls built with granite ashlars (**a**,**b**). Interior of Santa María Church, Guareña (Spain).

**Figure 11:**
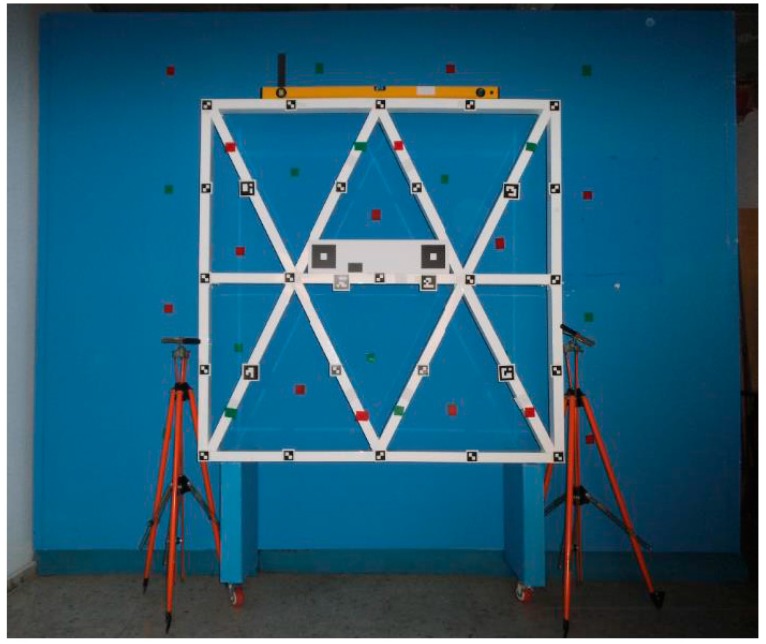
Aluminum structure used in the second experiment placed in front of the blue wall.

In order to perform the analysis of the accuracy of the EM in this mode of data capture, we have designed two experiments: the first, which was conducted inside a historic building that presents an optimal texture for the automatic correlation of images (Figure 10), and the second, which has the purpose of checking the operation of the equipment in unfavorable working conditions, has been carried out in premises where we have placed a metal aluminum structure, with a smooth, bright and white surface placed in front of a smooth blue wall and separated at a distance of 0.5 m (Figure 11).

**Figure 12:**
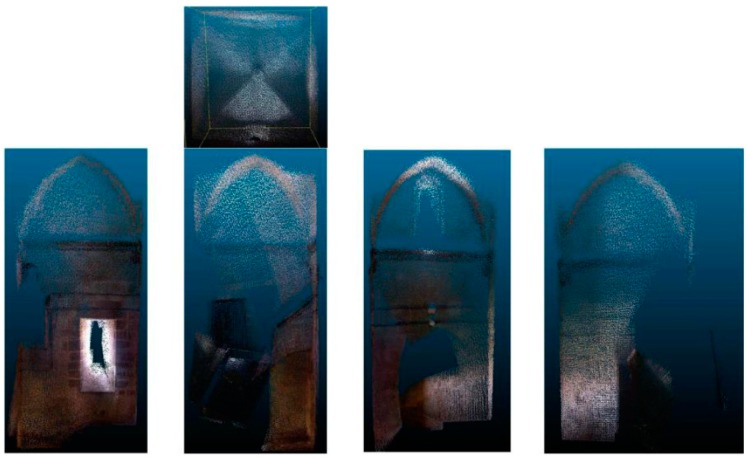
Point clouds obtained in the first experiment: different sections and ceiling.

Table 6 and the models of Figure 12 show the results obtained in the first experiment.

**Figure 13:**
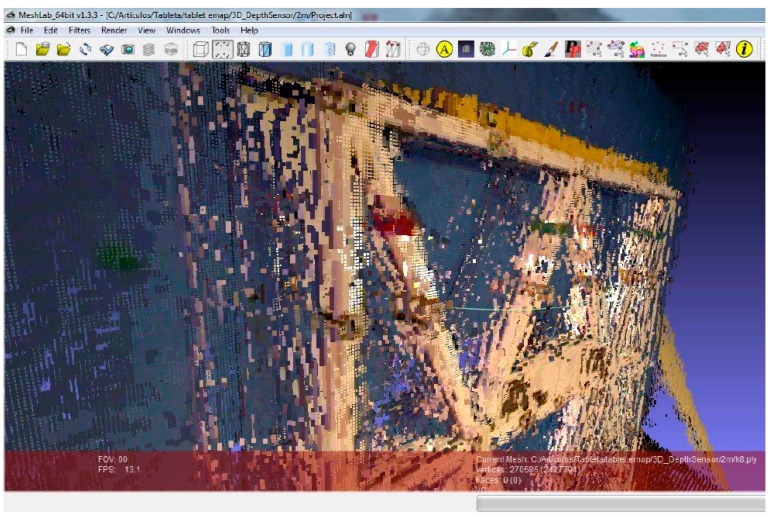
Erroneous point cloud obtained in the second experiment.

However, the tests conducted in the second experiment have not been successful because the resulting models are not acceptable (Figure 13) with these working conditions.

## 5. Conclusions

The tests show that precisions indicated by the EM manufacturer are greater than those obtained. Likewise, errors could not be quantified for measurements exceeding four meters from the object to be measured, as it was impossible to identify the center of symmetrical targets.

Errors vary in the distance of separation when capturing data by means of EM, a key factor in the precision of measurements. Error obtained following GSA requirements for the BIM Guide for 3D Imaging [32] shows that measurements by Photogrammetry are suitable for urban design projects, room space measurement, historical documentation, renovation and above ceiling condition. The measurements obtained by 3D Photomodeling (outdoor) and 3D Modeling with Depth Sensor (indoor) are conducive to level 1 projects for urban design and historical documentation.

Nonetheless, to reduce this error, an algorithm within the software for automatic recognition of the center of symmetrical targets or singular homologous points that serves to take some measurements is proposed. In this way, the estimated error produced by the operator would be minimized.

In addition, an error report that comments on the adjustment of photogrammetric models is recommended prior to obtaining the coordinates by Photogrammetry or the cloud points using 3D Photomodeling. Thus, the user would know whether the dimension of error in the photogrammetric adjustment is acceptable when performing a particular task.

Furthermore, it would be convenient for EM to report on what parameter values were used for internal, relative and absolute orientation for each picture once the adjustment has been made. In this sense, EM should also enter the precise value of these parameters. Thus, a user can resume a working session without having to start the entire process of adjusting each picture. Users could even work with historical pictures where orientation parameters were known.

Finally, the convenient portability of EM and its calculation of error make it complementary to the Scanstation, particularly with measurements difficult to obtain by the latter device.

## Figures and Tables

**Table 1:** EyesMap (EM) precision specified by the manufacturer.

Range	Accuracy STD ^1^	Accuracy STD Optimized Scale
3 m	3 mm	2.6 mm
15 m	15 mm	11 mm
30 m	30 mm	23 mm

^1^ Standard deviation (STD).

**Table 2:** Accuracy assessment result for photogrammetry data measured from 3 m to measured object (unit: mm).

Point ID	Error Vector *X*	Error Vector *Y*	Error Vector *Z*	Error
7	3	0	0	3
9	−10	0	−3	10
11	−9	0	−2	9
13	15	2	4	16
16	−6	−4	−2	7
18	−14	3	−5	15
20	−7	3	−2	8
22	−13	2	−3	13
25	−15	1	−1	15
27	12	−3	−4	13
29	11	2	3	12
31	12	1	3	12
34	12	1	3	12
Average error				11
RMSE	11	2	3	12
SAS				13

**Table 3:** Accuracy assessment result for 3D Photomodeling data measured from 3 m to measured object (unit: mm).

Point ID	Error Vector *X*	Error Vector *Y*	Error Vector *Z*	Error
3	12	−1	5	13
7	31	−2	−5	31
9	18	−2	5	19
11	−2	5	6	8
16	−4	12	8	15
18	16	0	0	16
20	16	0	0	16
22	0	8	2	8
25	0	5	1	5
27	19	2	−3	20
29	−5	5	0	7
32	−69	5	6	69
34	−11	7	−9	16
36	−5	7	1	8
Average error				18
RMSE	23	5	5	24
SAS				27

**Table 4:** Estimation of the standard deviation of the measurements for the targets. Distance: 3 m (unit: mm).

Point ID	Error Vector *X*	Error Vector *Y*	Error Vector *Z*	Error
4	13	17	−4	25
5	45	−3	−16	48
7	16	−4	−11	19
8	6	−5	−3	9
11	12	2	−35	38
12	25	4	−5	26
13	41	6	−2	41
18	25	17	−1	30
20	39	20	5	44
21	16	26	−6	31
24	−26	25	2	36
26	19	24	5	31
28	9	47	2	48
29	6	37	7	38
30	9	39	2	40
31	5	38	1	38
$STD_{T}$				11

**Table 5:** Relation between errors of measurements by Photogrammetry, 3D Photomodeling and the estimated error of the operator (unit: mm).

Distance (Meters)	${\mathrm{STD}}_{\mathrm{PH}}$	${\mathrm{STD}}_{\mathrm{CP}}$	${\mathrm{STD}}_{\mathrm{OP}}$	$\frac{STD_{OP}}{STD_{PH}}$	$\frac{STD_{OP}}{STD_{CP}}$
3	11	16	10	91%	62%

**Table 6:** First experiment. Results obtained with 17 control points (unit: mm).

	Error Vector *X*	Error Vector *Y*	Error Vector *Z*	Error
Average error				22
RMSE	16	11	11	22
SAS (90% probability)				32
